# Behavioral management of headache in children and adolescents

**DOI:** 10.1186/s10194-016-0671-4

**Published:** 2016-09-05

**Authors:** Noemi Faedda, Rita Cerutti, Paola Verdecchia, Daniele Migliorini, Marco Arruda, Vincenzo Guidetti

**Affiliations:** 1 Department of Pediatrics and Child and Adolescent Neuropsychiatry, Sapienza University of Rome, Via dei Sabelli, 108, 00185 Rome, Italy; 2 Department of Dynamic and Clinical Psychology, Sapienza University of Rome, Rome, Italy; 3 Department of Computer, Control and Management Engineering Antonio Ruberti, Sapienza University of Rome, Rome, Italy; 4 Glia Institute, Ribeirão Preto, São Paulo Brazil

**Keywords:** Headache, Behavioral therapies, Non-pharmacological therapies, Cognitive behavioral therapy, Children, Adolescents

## Abstract

Headache is the most frequent neurological symptom and the most prevalent pain in children and adolescents, and constitutes a serious health problem that may lead to impairment in several areas. Psychosocial factors, social environment, life events, school and family stressors are all closely related to headaches. A multidisciplinary strategy is fundamental in addressing headache in children and adolescents. Applying such a strategy can lead to reductions in frequency and severity of the pain, improving significantly the quality of life of these children.

It has been demonstrated that behavioral intervention is highly effective, especially in the treatment of paediatric headache, and can enhance or replace pharmacotherapy, with the advantage of eliminating dangerous side effects and or reducing costs. Behavioral interventions appear to maximize long-term therapeutic benefits and improve compliance with pharmacological treatment, which has proven a significant problem with child and adolescent with headache.

The goal of this review is to examine the existing literature on behavioral therapies used to treat headache in children and adolescents, and so provide an up-to-date picture of what behavioral therapy is and what its effectiveness is.

## Introduction

Headache affects about 60 % of children and adolescents all over the world. Tension type headache (prevalence of 20–25 %) is the most common cause of primary headache, followed by migraine (prevalence of 8 %) [1]. From epidemiology, it is clear that the onset of primary headache is often in childhood or adolescence [2] and the prevalence grows with children’s age. Research shows that children, adolescents and adults with headache do not seek medical care for their headaches and nearly half never receive a diagnosis [3].

Headache can affect all aspects of a child’s functioning. It has been associated with psychiatric illness, such as anxiety and depression, as well as psychosocial problems (e.g. school absences, problematic social interactions) [4, 5]. Recognizing anxiety, depressive symptoms and other psychiatric disorders should be considered in the clinical assessment of headache patients, especially in children and adolescents, as early identification of these symptoms may lead to improved headache management [6]. Furthermore the comorbidity with affective disorders and psychopathology in general may also enhance suicidal risk in migraine patients, especially in children and adolescent population, with a significant impact on public health [7]. Some studies investigated the gene-environment interaction and other risk factors involved in suicidal behaviour in headache patients [8, 9] showing that shared mechanism could be implicated in the pathophysiology of both migraine and affective disorders.

Furthermore an excessive use of medication in children and adolescents, and its association with certain undesirable behaviors or lifestyles, suggest educational initiatives on drug use are an important part of treatment [10]. Some non-pharmacological therapies seem to have effects that are similar to those of most drugs used for the prevention of migraine and tension-type headaches. These therapies often do not have dangerous adverse effects and are much less expensive than pharmacological therapies both in children and adolescents than in adults [11, 12].

An analysis of Mazzotta et al. [13] about the direct and indirect costs of headache in childhood and adolescence, indicates an overall cost of €18,614.30 for the treatment of twenty-five patients (7–18 years). The total direct cost is €17,290, of which 93 % covers visits and diagnostic testing, and the remaining 7 % covers symptomatic or prophylactic pharmacological therapy. The average expense for each patient is €691.60. In considering the different types of headaches, the study shows that the greatest expense is associated with Migraine with aura (MA) patients (€802.80 per patient), followed by chronic migraine (CM) patients (€760.50 per patient) and finally headache secondary to sinusitis patients (€692 per patient). The conclusion of the study of Mazzotta et al. [13] is that by combining effectiveness, minimal side effects, and cost savings, behavioral interventions provide a treatment option that can enhance or if necessary, replace pharmacotherapy.

Moreover, medications should generally not be prescribed alone but rather in combination with non-pharmacological therapies [14]. In addition, behavioral therapy help maximize long-term therapeutic benefit and ensure compliance with pharmacological treatment, which has been proven a significant problem with headache patients [15].

## Review

### Search strategy and selection criteria

We included the following search terms “headache” OR “migraine” and related terms, AND “behavioral management”, “behavioral treatment”, “behavioral therapy”, “non-pharmacological treatments”, “psychiatric comorbidities”, “cognitive-behavioral therapy”, “treatment adherence”, “psychological intervention”, “lifestyle habits” IN “children”, “childhood”, “child”, “adolescents”, “adolescence”, “young” and entered them into Pubmed databases. We reviewed the reference lists of paper identified and included articles judged as relevant. Highly regarded books published and additional non-peer-reviewed literature were included in this review, identified through a Google search and from citations in several key articles. We did our search between Sept 20, 2015, and June 15, 2016.

### What is behavioral therapy?

Behavioral treatment strategies for pain management have been researched intensively in recent years and are mostly derived from cognitive-behavioral therapy (CBT) [16] (Fig. ﻿1). In migraine treatment, behavioral treatment strategies have in many cases been shown to be as effective as pharmacological treatment [17], not only for headache management, but also to maintain a lifetime response to the headache treatment [15].

**Fig. 1:**
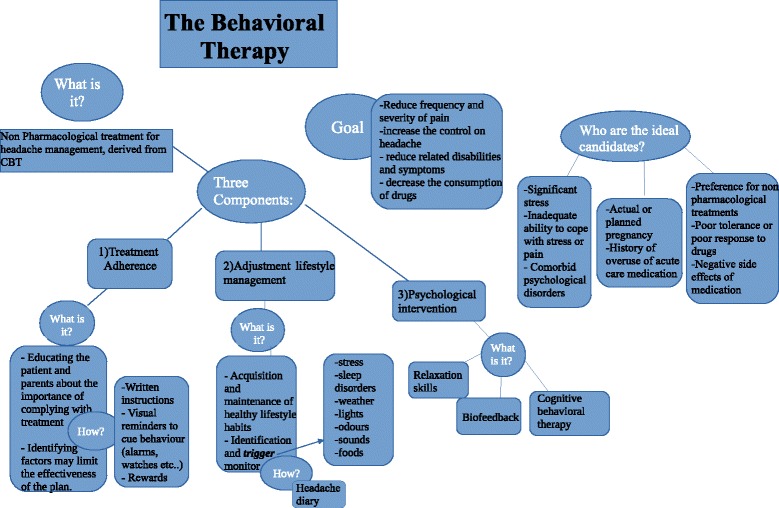
The behavioral therapy

The American Academy of Neurology–U.S. Consortium [18] noted that, given the presence of specific factors, behavioral and other non-pharmacological treatment are preferred to pharmacological treatment for migraine headache. These factors are:

- patient preference for non-pharmacological treatments;
- poor tolerance or poor response to pharmaceuticals;
- negative side effects of medication;
- actual or planned pregnancy;
- history of overuse of acute care medication;
- significant stress;
- inadequate ability to cope with stress or pain;
- comorbid psychological disorders [19]

In other words, individuals with clinical depression or anxiety, those with moderate-to-severe headache-related disabilities, those with difficulty managing triggers (including stress) or with other significant psychological issues (e.g., history of abuse/maltreatment), and those that prefer behavioral approaches are all ideal candidates for behavioral intervention [18, 20, 21].

The goals of behavioral treatments are to reduce the frequency and severity of pain, increase the patients’ control of their headaches, reduce related disabilities and symptoms, and limit reliance on poorly tolerated or unwanted medications [22].

The behavioral approach maximizes adherence to the prescribed headache treatment regimen, and incorporates the assessment of the impact of headache on a the child or adolescent’s quality of life, disability, and emotional functioning [23]. Non-adherence to prescribed treatment is an important and widespread behavioral health issue in the management of headache [24]. Poor adherence to prescribed treatment regimens can compromise the efficacy of medical treatments, and the health and quality of life of patients [25].

**Fig. 2:**
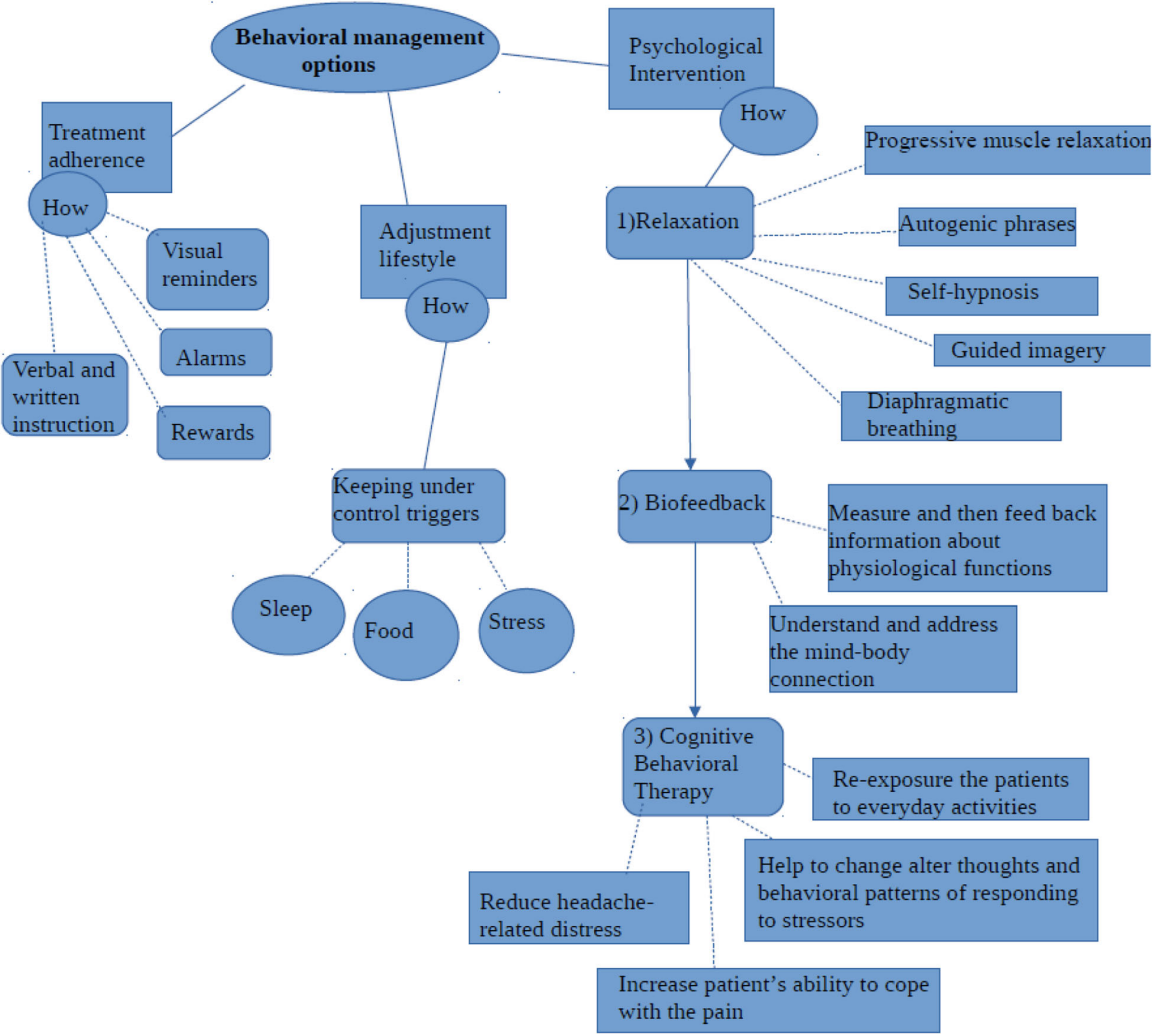
Behavioral management options

Behavioral therapy consists of three components [26] (Fig. 2):

1. Treatment adherence;
2. Adjustment of lifestyle management;
3. Psychological intervention.

#### Treatment adherence

The first component involves educating the patient and his or her parents about the importance of complying with the assigned treatment, and identifying factors may limit the effectiveness of the plan. Several studies support that non-adherence to treatment is an issue of utmost importance in the treatment of headache; indeed a good adherence to treatment has a positive impact on disease severity, risk of relapse, greater health care utilization, and sub optimal symptom management [24, 25]. The literature about this topic in children and adolescents is poor: to date a few studies have investigated this factor in pediatric headache [27–30].

Non-adherence to headache treatment is estimated at 50–88 % among children and adolescence [24, 31]. Treatment non-adherence can affect not only the prognosis of the disorder, with a worsening condition but also the overall health care system with an increased costs and an overuse of the health care system. Indeed non adherence to treatment leads to poor outcomes, which then increase service utilization and health care cost [32]. The factors that may limit the adherence to treatment can be divided in three categories: (1) regimen characteristics (e.g. difficulty of lifestyle changes), (2) disease characteristics (e.g. younger age of onset, frequency and severity of headache attacks), and (3) patient or family characteristics (e.g., premorbid/comorbid behavioral, psychopathology or dysfunctional family) [22, 33]. Factors that can have a positive effect on adherence are positive family functioning, close friends, internal locus of control, treatment with immediate benefits, and a good collaboration between doctor and patient [34]. The identification of needs, anxieties and expectations of the patients and their families regarding the treatment, allows the medical staff to monitor any possible preoccupation and fear, so that the best migraine management approach is administered to improve the quality of life of children and adolescents [35]. Interventions can have an educational, behavioral and clinical focus. Educational strategies provide verbal and written instructions to patients and their parents about the management of headache symptoms and the importance of the proposed treatment [22]. Educating patients can help reduce the helplessness and fear commonly experienced with migraine, enabling them to take control, reduce acute headache pain, and optimize long-term quality of life [36, 37]. Visual reminders to cue behaviour, such as charts for relaxation practice, alarms on watches or phones, self-monitoring, and rewards for treatment compliance are often used to help encourage adherent behaviour [22]. In children and adolescents a useful technique to remember is to pair a new behavior with a behavior that is already well-established in daily routine, such as brushing teeth [34]. Furthermore, a number of emotional and behavioral problems can occur, that interfere with the success of a treatment. These cases require clinical intervention strategies to modify these behavioural and emotional difficulties. All the obstacles, fears, anxieties and troubles that patients meet along the way, must be identified and addressed in the plan.

#### Adjustment of lifestyle habits

Lifestyle interventions are focused on the acquisition and maintenance of healthy lifestyle habits [22]. Clinicians have the task of assisting children and their families in changing lifestyle habits, and of pointing out the importance of this issue. Indeed, lifestyle habits have a great influence on medical outcomes and changing unhealthy habits and behaviours can significantly improve quality of life [38]. There is evidence that unhealthy lifestyle habits are a trigger for childhood headaches [22, 26]. Headache triggers are varied and may have different results at different times, so it can be very difficult to identify them. A headache diary can be a useful way to keep headache attacks under control [39]. Headache triggers often include stress, sleep disorders, weather, lights, odours, sounds and specific foods [40–42].

The most common trigger was stress which was reported by 75.5 % of patients [41]; sources of stress in children and adolescents can include:

- school: new school or new teacher, tests and exams, bullying, learning disabilities
- family: very permissive or very strict parents, a new brother or sister, divorce, death of a family member.
- friends: very few friends, peers make fun of him/her.

Many studies show that there is a clear association between headache and sleep disorders [43, 44].

In sample of Neut D. et al., lack of sleep was reported by 69.6 % of patients [41]. Children with headache report more daytime symptoms of sleep disturbance including fatigue, tiredness, and sleepiness [45]. Bruni et al. [46] revealed that keeping a consistent bedtime and waking time, sleeping the same amount of hours every night, ensuring that sleep is continuous without frequent awakening, and restricting the intake of food and fluids prior to sleeping reduced headache frequency and duration.

Another commonly reported headache trigger in children are fluctuations in weather [40, 47]. Connelly et al. [47], found that relative humidity and presence of precipitation were significantly predictive of new headache onset in children and adolescents.

An abnormal sensitivity to or intolerance of light (photophobia) is reported as a trigger by 52.9 % of children and adolescents with headache [41]; a hypersensitivity to odours (osmophobia) by 55 % and an intolerance or hypersensitivity to sound (phonophobia) by 47 % [48]. These diseases affect mainly children with diagnosis of migraine than tension-type headache, so the presence of these factors can be a supportive diagnostic criterion for the differential diagnosis between migraine and tension-type headache [49].

Some foods can be a trigger for headache attacks, maybe because they activate biochemical mechanisms involved in headache disorder. For example, chocolate was reported by 11.8 % and cheese by 3.9 % [41]. On the other hand, the consumption of other food and drink items was found to be beneficial [50]. Nutraceuticals, for instance, can be effective in treating migraines in children and adolescents [51]. Moreover, magnesium, riboflavin, coenzyme Q10, the herbal extracts of butterbur, feverfew, and ginkolide B have all been suggested as preventatives for migraine [52–55]. Research has indicate that magnesium in migraine patients is significantly lower than the normal population, and is related to the frequency of migraine attacks, supporting the use of magnesium in prevention and treatment of migraine [56]. Foods that are high in magnesium include beans, fish, walnuts, rice, almonds, dark leafy greens, seeds, whole grains, avocados, yoghurt, bananas and dark chocolate. Riboflavin (B2), acting on mitochondrial metabolism, is shown to be effective in the treatment of migraines, even if more research is needed in children [57]. Coenzyme Q10 has demonstrated the potential to modify inflammatory changes and alteration of mitochondrial function that may occur during recurrent headaches [58]; foods that are also high in Q10 include cereals, nuts and vegetables. Combinations of ginko, coenzyme Q10, riboflavin, and magnesium have been studied in children and adolescents with migraine with positive outcomes [52, 59].

Researchers at the City of London Migraine Clinic found that feverfew a traditional medicinal herb, eliminates about two-thirds of migraines in a selected group of adult headache patients, which is similar to the effectiveness of most migraine drugs, [60] but to date there are no trials that indicate the safety or efficacy of feverfew for pediatric headache [61].

Oxidative stress, a disturbance in the balance between the production of free radicals and antioxidant defenses [62], plays a role in the development of paediatric migraine [63], so it is important finding new therapeutic factors with antioxidant properties for treatment of headache.

So there is evidence that lifestyle changes such as healthy sleeping habits, good hygiene, diet free of food additives are associated with a significantly lower frequency of migraine attacks and shorter disease duration for children and adolescents with migraine [64].

#### Psychological intervention

Psychological treatments are essential elements of the multidisciplinary, bio-psychosocial management of primary headache disorders, particularly for those with frequent or chronic headache, a high level of headache-related disability, medication overuse, or comorbid psychiatric symptoms [65]. In children and adolescents, headache is commonly associated with several psychiatric comorbidities, in particular depression, anxiety and attention-deficit/hyperactivity disorder [5]. Teaching relaxation techniques, stress reduction, increasing physical activity, and other psychological interventions should be considered standard management options for children with headache and other comorbidities [36]. Self-regulation strategies, such as relaxation and biofeedback, as well as cognitive behavior therapies are reported as the most commonly used behavioral treatments for headache [22, 66]. These treatments emphasize the active involvement of patients and they aim to change dysfunctional thoughts and maladaptive behaviors, in order to facilitate the use of effective strategies for coping with pain, and to improve headache symptoms [67].

Active involvement of patients can lead to increased confidence in abilities to prevent and manage headaches [22, 68], which in turn can lead to less headache-related disability [22, 69]. In management of primary headache, psychological treatments include [65]:

- Relaxation skills;
- Biofeedback
- Cognitive behavioral therapy.

Relaxation skills are used to decrease headache by enabling patients to modify their own headache-related physiological responses and decrease sympathetic arousal [22, 70]. Techniques to reduce tension include: progressive muscle relaxation (PMR), autogenic phrases, self-hypnosis, guided imagery (GI), and diaphragmatic breathing [71] (Table 1). These techniques have been shown to be as effective as pharmacological treatment in child, adolescent and adult, improving the frequency, intensity, and duration of headache [72, 73].

**Table 1:** Relaxation skills

Relaxation skill	What is it?
Progressive muscle relaxation	Series of relaxation exercises
Autogenic phrases	Phrases focused on several parts of the body (e.g. “my arms are heavy”)
Self-hypnosis	Process of self-induced hypnosis
Guided imagery	Patients must focus on image of peace and serenity
Diaphragmatic breathing	Patients learn to breath using diaphragm

Biofeedback refers to the use of electronic or electromechanical tools to measure and then feed back information about physiological functions and it is useful in paediatric headache to understanding and addressing the mind-body connection [22, 74]. Multiple published studies have suggested that biofeedback is effective in reducing the frequency and severity of headaches, often allowing patients to decrease their dependence on medication [75].

Cognitive behavioral therapy is a treatment that targets behaviours, emotions, and cognitions that trigger or aggravate headaches [66]. CBT consists mainly of cognitive and behavioral techniques and it helps the patient alter thoughts, interpretations of events, assumptions, and typical behavioral patterns of responding to stressors or events, increasing patient’s ability to cope with the pain and to reduce headache-related distress [22]. Cognitive-behavioral techniques, in particular, have been found to reduce the intensity and frequency of headache in children and adolescents [76]. It is proposed that these competencies could contribute to the successful long-term prevention of an adverse course of headache into adulthood [77].

### The Efficacy of Behavioral Intervention in children and adolescence with headache

Behavioral interventions provide a treatment option that can enhance or if necessary, replace pharmacotherapy. The combination of both pharmacological and non-pharmacological treatment has been shown to be superior to each individually, and appears to maximize long-term therapeutic benefit of treatment [78, 79]. During the past several decades, it has become clear that all mental processes derive from mechanisms of the brain [80]. This means that any change in our psychological processes is reflected by changes in the functions or structures of the brain and there is clear evidence that our subjective experiences affect the brain [81]. Psychotherapy provides the learning of new alternative ways of thinking and behaving. This learning can bring change in the brain by altering the strength of synaptic interactions between neurons, thereby leading to real morphological changes in the neurons themselves and generating new conditions in the brain [82, 83].

Neuroimaging studies have found that psychotherapy has measurable effects on the central nervous system: psychotherapy may modify brain function and metabolism in specific brain areas [84, 85]. Many psychotherapies attempt to enhance patients’ problem-solving capacities, self-representation, and regulation of affective states [86]. The brain areas involved in these functions are the dorsolateral prefrontal cortex, the ventral anterior cingulate cortex, the dorsal anterior cingulate cortex, the ventral and dorsal sub-regions of the medial prefrontal cortex, the posterior cingulate cortex, the precuneus, the insular cortex, the amygdala, and the ventrolateral prefrontal cortex [87]. About twenty studies have been published on brain effects of psychological treatments for obsessive compulsive disorder, anxiety disorder (e.g. panic disorder and social phobia), schizophrenia and depression. In this way it is possible to treat the comorbidities present in subjects with headache and thereby improve their quality of life.

Children with chronic physical illness are significantly more likely to develop common psychiatric symptoms than otherwise healthy children [88], and high levels of psychological comorbidity [5] has led to migraine becoming more commonly viewed as a bio-psychosocial condition, influenced by cognitive, emotional and environmental factors, as well as biological [89].

A recent systematic review about psychological interventions for mental health disorders in children with chronic physical illness, in fact, shows that children may benefit from cognitive behavioral interventions for depression and anxiety in the context of a comorbid chronic physical health problem [88].

A recent meta-analysis showed that psychological therapies can significantly reduce pain and disability in children and adolescents with chronic pain, and in particular headache pain [90]. Reviews using CBT on children, adolescents and adult headache patients showed reduction in migraine and tension-type headache attacks in a pre-/post-treatment for intensity, frequency, and duration of headache, significantly superior to control conditions [11, 66, 91–93].

Power et al. [12] carried out a study to determine the benefits of CBT in children and adolescents (aged 10–17) with chronic migraine, when combined with amitriptyline versus headache education plus amitriptyline. They find that, in children and adolescents with chronic migraine, the use of CBT plus amitriptyline is associated with greater reductions in days with headache and migraine-related disability compared with use of headache education plus amitriptyline. These findings support the efficacy of CBT in the treatment of chronic migraine in children and adolescents. Chen YZ et al. [94] investigate the preventive effect of behavioral therapy plus flunarizine in children with migraine, and report that preventive treatment of behavioral therapy plus oral flunarizine had better clinical efficacy than oral flunarizine alone, in children with migraine.

Relaxation techniques, including PMR, autogenic phrases, self-hypnosis, GI, and diaphragmatic breathing has been shown to be an effective therapeutic approach for the management of headaches in children and adolescents [27, 95–99]. Trautmann et al. [100] found that relaxation, biofeedback, and CBT are highly effective in treating headache symptoms, both in frequency and severity. Relaxation or biofeedback alone obtain improvement in about 50 %; when combined about 60 % improvement was seen [101]. With CBT and biofeedback, improvement in children with migraine reaches 75 % [102]. Blume et al., found a positive response to biofeedback in 58 % and a 43 % reduction in headache days with biofeedback therapy in children and adolescents with headache [103].

Meta-analytic findings of Palermo et al. demonstrated a large positive effect of psychological intervention on pain reduction at immediate post-treatment and follow-up in youth with headache. It would appear that cognitive-behavioral therapy, relaxation therapy, and biofeedback all produced improvement in pain relief [104].

Fisher et al. [105] show the efficacy also of psychological therapies delivered remotely, compared to waiting-list, treatment-as-usual, or active control treatments, for the management of chronic pain in children and adolescents. Psychological therapy delivered remotely confirms benefit in reducing the intensity or severity of pain in children.

Penzien et al. report [19] the following recommendations pertaining to behavioral interventions for migraine (as they have been outlined by the US Headache Consortium) [106]: (1) relaxation training, thermal biofeedback combined with relaxation training, electromyographic biofeedback, and cognitive behavioral therapy are treatment options for prevention of migraine (grade A evidence); and (2) behavioral therapy may be combined with preventive drug therapy to achieve added clinical improvement for migraine (grade B evidence).

Further studies are needed to point out the exact mechanisms of behavioral treatments [107] in children and adolescents with primary headache, which are likely multifactorial.

### Limitations

Behavioral therapy like other forms of headache management is imperfect [108], and some issues make it inadequate for some people (Table 2 Strengths and limitations):

1. Behavioral approaches give us an idea of how individuals react to specific life situations, but patterns of behaviour may vary over the course of an individual’s life [108].
2. Although behavioral treatment is considered a short-term therapy, it takes time to change same behaviours and replace negative behaviours with positive ones. Parents and teachers may find this frustrating.
3. Behavioral therapy has focused on the development and study of specific effective techniques to address different clinical situations; however, it sometimes does not always take into account individual differences that imply that these methods cannot be applied in the same way to all subjects [108].
4. Behavioral therapy teaches individuals some skills for coping with the problems (cognitive restructuring, problem solving, relaxation), but teaching parents skills to work more effectively with their children can result in a poor compliance. A working relationship between the patient and therapist is an essential part of any psychotherapy and is crucial for patient’s improvement [109].
5. In behavioral interventions the emphasis is on the “here and now” [110], but sometimes it is necessary to go back in time and explore the origin of the disorder.
6. Behavioral therapists must recognize that they have become embedded in a relationship with the patient, so they must maintain a sense of awareness and control over their own emotional reactions to the patient’s behaviour [111].
7. CBT may not be effective for people with severe mental disorders or for those with learning difficulties. The focus of CBT is always on patients and their capacity to bring change to themselves [112].

**Table 2:** Strengths and limitations

Strengths	Limitations
Cost saving	It takes time to change same behaviors, so parents and teachers may find this frustrating.
Ensure Compliance	Teach parents how work more effectively with their children can result in a poor compliance
Maximize long term therapeutic benefit	The focus is “here and now”
No negative side effects of medications	Therapist must maintain a sense of control and awareness
Learning of new alternative ways of thinking and behaving	Patterns of behavior may change over the course of life
Reduction of headache severity and frequency	It is not effective for people with severe mental disorder
Reduce pain and disability	It does not always take into account individual differences

Behavioral headache interventions continue to face significant challenges that in part derive from the inability to conduct a truly double blind trial, and from limited availability of behaviourally trained headache clinicians [19, 113]. Rains in his review about the “Behavioral Headache Treatment” [66] has presented a critique of methodological quality of the clinical trials literature, highlighting the strengths and weaknesses in relation to recruitment and selection of patients, sample size and statistical power, the use of a credible control, and the reproducibility of the study interventions in clinical practice. There is a little research on the effectiveness of behavioral headache interventions in children and adolescence and this topic has often been discussed rather than clinically investigated.

## Conclusion

Headache is a common condition among children and adolescents that has a large impact on school and other areas of daily life, and more generally on the quality of life of children and their families. Lacking proper care, many children will continue to experience headaches into adulthood. These considerations point to the importance of prompt, effective, and early intervention for paediatric headache [4].

Multidisciplinary treatment is an effective strategy for children and adolescents and shows improvement in multiple measures of outcome, including frequency and severity of headache and number of school days missed because of headache. Behavioral therapy is very useful to such an intervention [114]. Attention to the effects of headache, to needs and fears of children and adolescents, and to factors that may limit the effectiveness of the therapy, is an important part of treatment.

Behavioral therapy may reduce the need for medication, and help maintain effects over the long term. There is evidence that psychological treatments are effective in reducing pain intensity in children and adolescents with headache, and that therapies such as relaxation and cognitive behavioral therapy may have lasting effect in improving mood and reducing pain for chronic headache [115]. The application of behavioral therapy in the management of headache in children and adolescents is an effective alternative to drugs without the problematic and dangerous side effects of pharmacological treatments and the cost savings of not having to purchase prescription or over-the-counter medications [116, 117]. Clinicians should inquire about behavioral therapy and other non pharmacological treatments for migraine, in order to plan an integrative approach, especially in children and adolescents with headache.

## Abbreviations

CBT: Cognitive-behavioral therapy
CM: Chronic migraine
GI: Guided imagery
MA: Migraine with aura
PMR: Progressive muscle relaxation
